# Comparison of distal radius fracture plating surgery under wide-awake local anesthesia no tourniquet technique and balanced anesthesia: a retrospective cohort study

**DOI:** 10.1186/s13018-023-04243-0

**Published:** 2023-10-03

**Authors:** Chih-Ting Chen, Shih-Hsiang Chou, Hsuan-Ti Huang, Yin-Chih Fu, Jesse B. Jupiter, Wen-Chih Liu

**Affiliations:** 1 Department of Clinical Education, Kaohsiung Medical University Hospital, Kaohsiung, Taiwan; 2 Department Orthopedic Surgery, Kaohsiung Medical University Hospital, Kaohsiung, Taiwan; 3 Orthopedic Research Center, Kaohsiung Medical University, Kaohsiung, Taiwan; 4 Department of Orthopedic Surgery, Kaohsiung Municipal Ta-Tung Hospital, Kaohsiung, Taiwan; 5 College of Medicine, Kaohsiung Medical University, Kaohsiung, Taiwan; 6 Regeneration Medicine and Cell Therapy Research Center, Kaohsiung Medical University, Kaohsiung, Taiwan; 7 Hand and Arm Research Center, Department of Orthopedic Surgery, Massachusetts General Hospital, Harvard Medical School, Boston, USA; 8 School of Medicine, College of Medicine, Kaohsiung Medical University, Kaohsiung, Taiwan

**Keywords:** WALANT, Wide-awake local anesthesia no tourniquet, Wide-awake hand surgery, Distal radius fracture

## Abstract

**Background:**

Distal radius fractures (DRF) are frequently treated with internal fixation under general anesthesia or a brachial plexus block. Recently, the wide-awake local anesthesia with no tourniquet (WALANT) technique has been suggested as a method that results in higher patient satisfaction. This study aimed to evaluate the functional outcomes, complications, and patient-reported outcomes of DRF plating surgery under both the WALANT and balanced anesthesia (BA).

**Methods:**

Ninety-three patients with DRFs who underwent open reduction and plating were included. Regarding the anesthetic technique, 38 patients received WALANT, while 55 received BA, comprised of multimodal pain control brachial plexus anesthesia with light general support. The patient's overall satisfaction in both groups and the intraoperative numerical rating scale of pain and anxiety (0–10) in the WALANT group were recorded. The peri-operative radiographic parameters were measured; the clinical outcomes, including Quick Disabilities of the Arm, Shoulder, and Hand (*Quick*DASH) score, wrist mobility, and grip strength, were recorded in up to 1-year follow-up. Results presented with a mean difference and 95% confidence intervals and mean ± standard deviation.

**Results:**

The mean age of patients in the WALANT group was higher than in the BA group (63 ± 17 vs. 54 ± 17, *P* = 0.005), and there were fewer intra-articular DRF fractures in the WALANT group than in the BA group (AO type A/B/C: 30/3/5 vs. 26/10/19, *P* = 0.009). The reduction and plating quality were similar in both groups. The clinical outcomes at follow-up were comparable between the two groups, except the WALANT group had worse postoperative 3-month pronation (88% vs. 96%; − 8.0% [ − 15.7 to − 0.2%]) and 6-month pronation (92% vs. 100%; − 9.1% [ − 17.0 to − 1.2%]), and better postoperative 1-year flexion (94% vs. 82%; 12.0% [2.0–22.1%]). The overall satisfaction was comparable in the WALANT and BA groups (8.7 vs. 8.5; 0.2 [ − 0.8 to 1.2]). Patients in the WALANT group reported an injection pain scale of 1.7 ± 2.0, an intraoperative pain scale of 1.2 ± 1.9, and an intraoperative anxiety scale of 2.3 ± 2.8.

**Conclusion:**

The reduction quality, functional outcomes, and overall satisfaction were comparable between the WALANT and BA groups. With meticulous preoperative planning, the WALANT technique could be an alternative for DRF plating surgery in selected patients.

*Trial registration* This retrospective study was approved by the Institutional Review Board of Kaohsiung Medical University Hospital (KMUHIRB-E(I)-20210201).

**Supplementary Information:**

The online version contains supplementary material available at 10.1186/s13018-023-04243-0.

## Background

Distal radius fracture (DRF) is a common injury that can require surgical intervention. The use of wide-awake local anesthesia with no tourniquet (WALANT) has been investigated in several studies for DRFs [1], olecranon fractures [2], ankle fractures [3], and clavicle fractures [4] fracture surgeries, offering many advantages such as cost-effectiveness [5, 6], improved patient satisfaction [5], and reduced risks associated with systemic anesthesia [7].

As life expectancy increases and pursues a better quality of life, some elderly patients with comorbidities seek surgical treatments for quicker recovery and preserved functionality after injury. With the concept of balanced anesthesia (BA), comprised of multimodal pain control, has become popular and lowered the need for gas anesthetics and opioids [8]. However, a previous study found that the intraoperative hemodynamics was comparable among patients who underwent plating surgeries for DRF via BA and WALANT technique [9].

Previous studies have shown the effectiveness of surgical management for DRFs via the WALANT technique [1]. However, whether WALANT is suitable for all cases of DRF surgeries is still being determined. In real-world clinical scenarios, the characteristics of patients who chose WALANT might differ from those who chose GA and brachial plexus block.

In this study, we reviewed a cohort of patients with DRF undergoing plating surgeries via the BA or WALANT technique. The study aimed to investigate the patient’s characteristics, surgical outcomes, intraoperative pain, anxiety, and satisfaction. The results provided valuable information for surgeons to recognize the benefits and limitations of using WALANT as an alternative to BA.

## Methods

### Patient selection and group division

Ninety-three adults with DRFs who underwent open reduction and plating surgery were recruited at a university-affiliated hospital between August 2018 and August 2020. The Strengthening the Reporting of Observational Studies in Epidemiology guidelines were followed throughout this study. Regarding the anesthetic technique, 38 patients received WALANT, while 55 received BA. The choice of anesthesia method is a patient-shared decision-making process, depending on multiple factors, including the patient’s willingness, medical history, the severity of the injury, and the anticipated complexity of the surgical process. For instance, if a surgery necessitates multiple incisions and involves substantial injections of local anesthetics, it can lead to severe swelling in the wrist. Additionally, if the patient cannot tolerate lying flat for the duration of the surgery, it poses another challenge. We do not recommend employing the WALANT technique for these patients. The Institutional Review Board approved this retrospective cohort study. Patients aged over 20 with DRFs and who underwent plating surgery were included. The exclusion criteria were: 1. Age ≤ 20 years old, 2. Open fracture, 3. Pathological fracture, 4. DRF with other skeletal injuries. In the WALANT group, local anesthetics were administered by an orthopedic surgeon, whereas in the BA group, anesthetics were provided by an anesthesiologist. The same orthopedic surgeon performed all surgical procedures for both groups of patients.

### Anesthesia and surgical technique

**Fig. 1:**
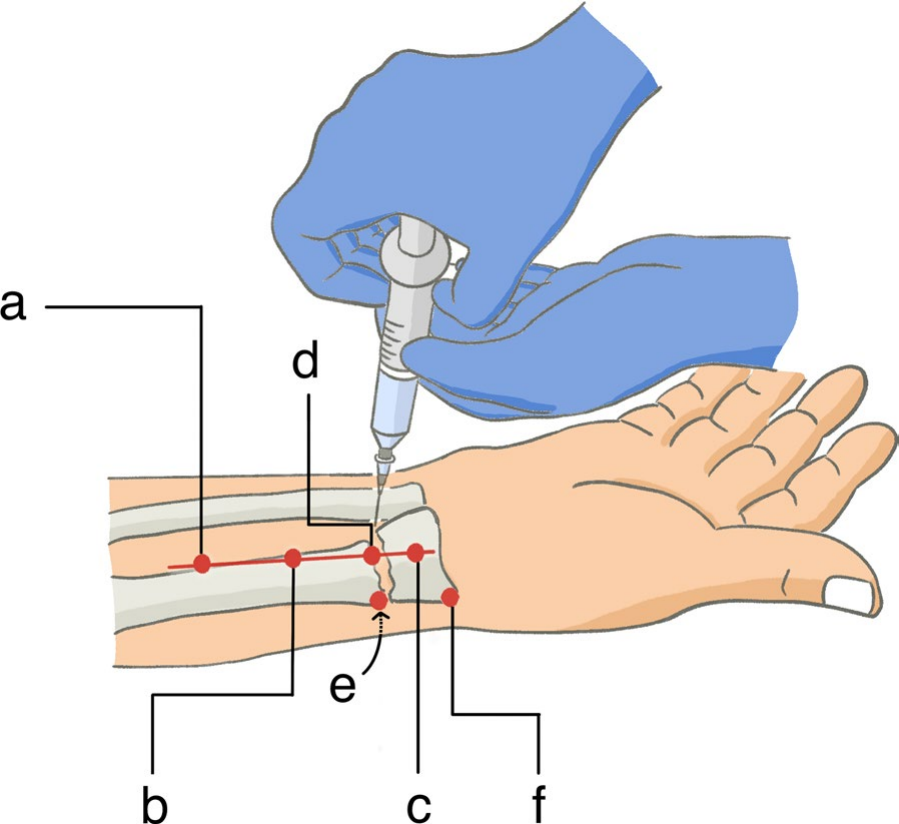
Schematic outline of local anesthetic injection. **a** 2–3 mL of 1% lidocaine was injected into the subcutaneous fat with a 26-G needle; **b** and **c** 15 mL of 1% lidocaine was injected along the planned volar incision with a 22-G needle; **d** 10 mL of 1% lidocaine was injected into the fracture site with a 24 G needle; **e** 10 mL of 1% lidocaine was injected along the dorsal periosteum; **f** 2–3 mL of 1% lidocaine was injected over the radial styloid to prepare for preliminary K-wire fixation

In the WALANT group, 1% lidocaine with 1:100,000 epinephrine was used with a conservative upper limit of 7 mg/kg [10]. For example, the maximum dose for a 60 kg patient was 420 mg (42 mL) of 1% lidocaine. The patients’ forearms were supine, and 2–3 mL of 1% lidocaine was injected into the subcutaneous fat using a 26-G needle. Next, the 26-G needle was exchanged with a 22-G long needle, and the local anesthetic was slowly injected along the planned volar incision from the same entry point. Typically, 15 mL of 1% lidocaine is sufficient to cover the entire volar surface of the subcutaneous area for the Henry approach [11]. A 24 G needle was used to penetrate the pronator quadratus and touch the volar surface of the radius, and 10 mL of local anesthetic was injected into the fracture site and along the volar periosteum of the distal radius. This step is referred to as the hematoma block [12]. Our approach diverges from the traditional hematoma block in that our objective was to infuse local anesthetic around the periosteum surrounding the fracture to reduce pain when the periosteum is irritated. We achieved this by inserting a 24G needle between the radial artery and the flexor digitorum radialis, a landmark that ensures the median nerve is located on its ulnar side. It is imperative to maintain negative pressure in the syringe throughout the entire needle insertion process to minimize the risk of vascular injury. Then, patients were asked to pronate their forearms, and 10 mL of local anesthetic was injected along the dorsal periosteum. Finally, 2–3 mL of 1% lidocaine was injected over the radial styloid to prepare for preliminary K-wire fixation (Fig. 1). The local anesthetic injection procedure typically took 5 min to perform. To allow for a better hemostatic effect, the incision would take place after 20–25 min waiting period [13]. The local anesthetic effect lasted approximately 6 h around the wrist [14].

In the BA group, patients received this standard approach in the senior author’s hospital. BA was induced with 1 mcg/kg fentanyl and 2 mg/kg propofol and maintained with 2%–4% sevoflurane via laryngeal mask airway. Optionally, the anesthesiologist provided a supraclavicular block with 25 mL of 0.25% bupivacaine under real-time ultrasound guidance. Without the local hemostatic effect of epinephrine, a tourniquet was applied to the patient’s upper arm. Surgery for DRF was performed after exsanguinating the forearm blood with an Esmach bandage and setting the tourniquet at 250 mmHg.

Considering the availability of instruments at different periods at our institution and the surgeon’s experience and preference in choosing specific fracture patterns with some plate designs, four different volar locking plates (ACU-LOC plate, ACUMED, LLC., USA; Anatomic Volar Plate System, DePuy Synthes, Johnson & Johnson Co., USA; Distal R.A.F. Locking plate, APLUS Co., Taiwan; DVR® plating system, Zimmer Biomet, USA) were used for internal fixation. In our study, both volar and dorsal approaches were used for fracture exposure based on the individual fracture pattern and the surgeon's judgment. Among the WALANT group, 36 patients (94.7%) underwent a volar approach, one (2.6%) underwent a dorsal approach, and one patient (2.6%) underwent both volar and dorsal approaches. In the BA group, 47 patients (85.5%) underwent a volar approach, six (10.9%) underwent a dorsal approach, and two (3.6%) underwent both approaches. The plate was applied after proper reduction of the fracture.

### Postoperative treatment

In the BA group, patients stayed overnight for observation; however, the WALANT group was discharged immediately from the hospital without staying at the post-anesthesia care unit (PACU). In both groups, a volar plaster splint was applied as a part of postoperative care; the plaster splint was converted to a removable splint at the first postoperative visit within one week postoperatively. After removing stitches, patients were advised to use therapy putty to facilitate digit flexion. As a general guideline, it is recommended not to use the injured hand for heavy activities for at least six weeks postoperatively. The senior author (W.-C.L.) paid special attention to finger flexion and wrist supination. The initiation of wrist motion exercises is determined based on various factors, including the severity of the fracture, bone quality, and analysis of follow-up X-rays. While most patients could exercise independently, those facing delays in digit flexion or wrist motion were referred to a hand therapist.

### Assessment

The patient’s age, sex, medical and surgical history were recorded, and the preparation time, operative time, surgical approach, and blood loss during surgery. The fracture patterns were classified based on the AO Foundation/ Orthopaedic Trauma Association (AO/OTA) [15]. Patient intraoperative experience (numerical rating scale of the pain and anxiety) and overall satisfaction were evaluated during the 1st or 2nd postoperative visits.

Intraoperative blood loss was estimated using the suction bottle, and estimated blood in gauze. Estimated blood loss in gauze was obtained by calculating the weight difference before and after the operation [16]. Immediate postoperative radiographic images were used to examine the quality of reduction and plating. The type of fracture, radial height, radial inclination, volar tilt, ulnar variance, and articular step-offs were compared between the two groups by two qualified orthopedic residents and confirmed by a senior hand surgeon. The above radiographic parameters were measured based on the methodology described in the literature [17]. Radial inclination (degrees) and ulnar variance (mm) were measured using the posteroanterior wrist view. The volar tilt (degrees) was measured on the lateral view. The articular step-off (cm) was determined based on the most significant articular gap in the posteroanterior or lateral view. Anesthesia-related and surgery-related complications were also recorded.

Clinical outcomes were evaluated at multiple time points post-surgery: 6 weeks, 3 months, 6 months, and 1 year. Outcome measures included the Quick Disabilities of the Arm, Shoulder, and Hand (*Quick*DASH) score, pain scale score, wrist mobility (in degrees), and grip strength (in kilograms). Patients in the BA group were also asked to complete a Single Assessment Numeric Evaluation (SANE) to rate their overall satisfaction with the surgery (on a scale of 0–10) [18]. To gain a better understanding of the intraoperative experience of patients in the WALANT group, we administered a patient experience survey consisting of several questions (Additional file 1). These questions included whether the patient had ever undergone surgery under general anesthesia or WALANT before, the level of pain experienced during anesthesia injection and surgery (on a scale of 0–10), the level of anxiety felt during surgery (on a scale of 0–10), whether any discomfort was experienced after the surgery, preference for anesthesia under sedation or WALANT if given a choice again (and the reason for that preference), and overall satisfaction with WALANT surgery (on a scale of 0–10).

### Statistical analysis

Continuous variables were expressed as the mean ± standard deviation, and categorical variables were expressed as the total number of events. Pearson chi-square was used to analyze categorical data. Student-t test was used to analyze continuous data. For the wrist mobilities, the effect size was presented as a mean difference (MD) of the percentage of the injured to the non-injured one; for the grip strength, the effect size was presented as MD of the injured wrist; for the *Quick*DASH score, the effect size was presented as MD of the scores. A 95% confidence interval (CI) for the effect size and the *P*-value were provided.

## Results

Ninety-three adults with DRFs who underwent open reduction and plating surgery were included (Table 1). In the WALANT group, AO/OTA classification 2R3A, 2R3B, and 2R3C fractures corresponded to 78.9%, 7.9%, and 13.2% of the cases, respectively. In the BA group, AO/OTA classification 2R3A, 2R3B, and 2R3C fractures corresponded to 47.3%, 18.2%, and 34.5% of the cases, respectively. The mean surgical time was shorter for the WALANT group than for the BA group (71.5 vs. 91.3 min; MD, − 19.8 [ − 33.8 to − 5.9]; *P* < 0.01).

**Table 1:** Demographic data

	WALANT	BA	*P* value
*Patient number*	38	55	
*Age (year)*	63.0 ± 16.5	54.4 ± 16.7	**0.007**
*Sex*			
Male	8	22	0.072
Female	30	33	
*Comorbidity*			
Diabetes Mellitus	6	8	1.000
Hypertension	11	9	0.200
Cerebral vascular accident	4	1	0.155
End-stage renal disease	3	0	0.065
Chronic heart failure	1	0	0.409
*Fracture classification*			
AO/OTA 2R3A	30	26	**0.009**
AO/OTA 2R3B	3	10	
AO/OTA 2R3C	5	19	
*Blood loss (ml)*	23.4 ± 32.7	10.0 ± 16.8	**0.010**
*Operation time (minute)*	71.5 ± 24.0	91.3 ± 43.2	**0.012**
*Complication*			
Infection	0	0	–
Neuropraxia	0	0	–
Compartment syndrome	0	0	–
Perioperative cardiovascular event	0	0	–
Hand vascular compromise event	1	0	0.409
Exchange to general anesthesia	1	–	–

WALANT, wide-awake local anesthesia no tourniquet; BA, balanced anesthesia; Mean ± Standard deviation; Bold means *P*-value < 0.05

### Additional operations and complications

Comorbidities were similar in both groups, but two patients in the WALANT group had arteriovenous fistulas for hemodialysis. Postoperative radiographs showed similar reduction and plating quality between the groups (Table 2). No perioperative cardiovascular events, infection, neuropraxia, or compartment syndrome were observed. However, transient cyanosis and vascular compromise were noted in the WALANT group, with no permanent complications during follow-up [19]. An 82-year-old woman who had previously undergone open reduction and internal fixation of her left DRF via the WALANT technique broke her right wrist two years later and chose to undergo the same procedure again. Unfortunately, a peri-implant fracture occurred because of severe osteoporosis. As the maximum dose of lidocaine had already been administered, the surgeon converted the procedure to BA and used a longer plate with an extended wound.

**Table 2:** Radiographic parameters before and after surgery

	Pre-surgery				Post-surgery			
	WALANT	BA	MD (95% CI)	*P* value	WALANT	BA	MD (95% CI)	*P* value
Radial inclination (°)	13.0 ± 8.4	15.7 ± 6.3	− 2.7 ( − 5.8 to 0.4)	0.09	20.9 ± 4.1	20.4 ± 3.8	0.5 ( − 1.1 to 2.2)	0.53
Radial height (mm)	5.9 ± 3.8	7.4 ± 3.1	− 0.1 ( − 0.3 to 0.0)	0.06	9.5 ± 2.0	9.4 ± 1.9	0.0 ( − 0.1 to 0.1)	0.75
Ulnar variance (mm)	1.8 ± 3.0	2.3 ± 2.4	− 0.1 ( − 0.2 to 0.1)	0.35	0.4 ± 2.2	− 0.4 ± 1.5	0.1 (0.0 to 0.2)	0.03
Articular step-off (cm)	0.17 ± 0.47	0.41 ± 0.89	0.24 ( − 0.07 to 0.55)	0.43	0.04 ± 0.21	0.08 ± 0.22	0.04 ( − 0.05 to 0.13)	0.45
Volar tilt (°)	− 9.0 ± 22.2	− 5.3 ± 21.9	− 3.7 ( − 13.5 to 6.1)	0.45	8.5 ± 7.9	8.5 ± 8.6	1.8 ( − 3.5 to 3.5)	1.00

Mean ± Standard deviation; MD, mean difference; CI, confidence interval; WALANT, wide-awake local anesthesia no tourniquet; BA, balanced anesthesia

### Function and patient-reported outcomes

**Fig. 2:**
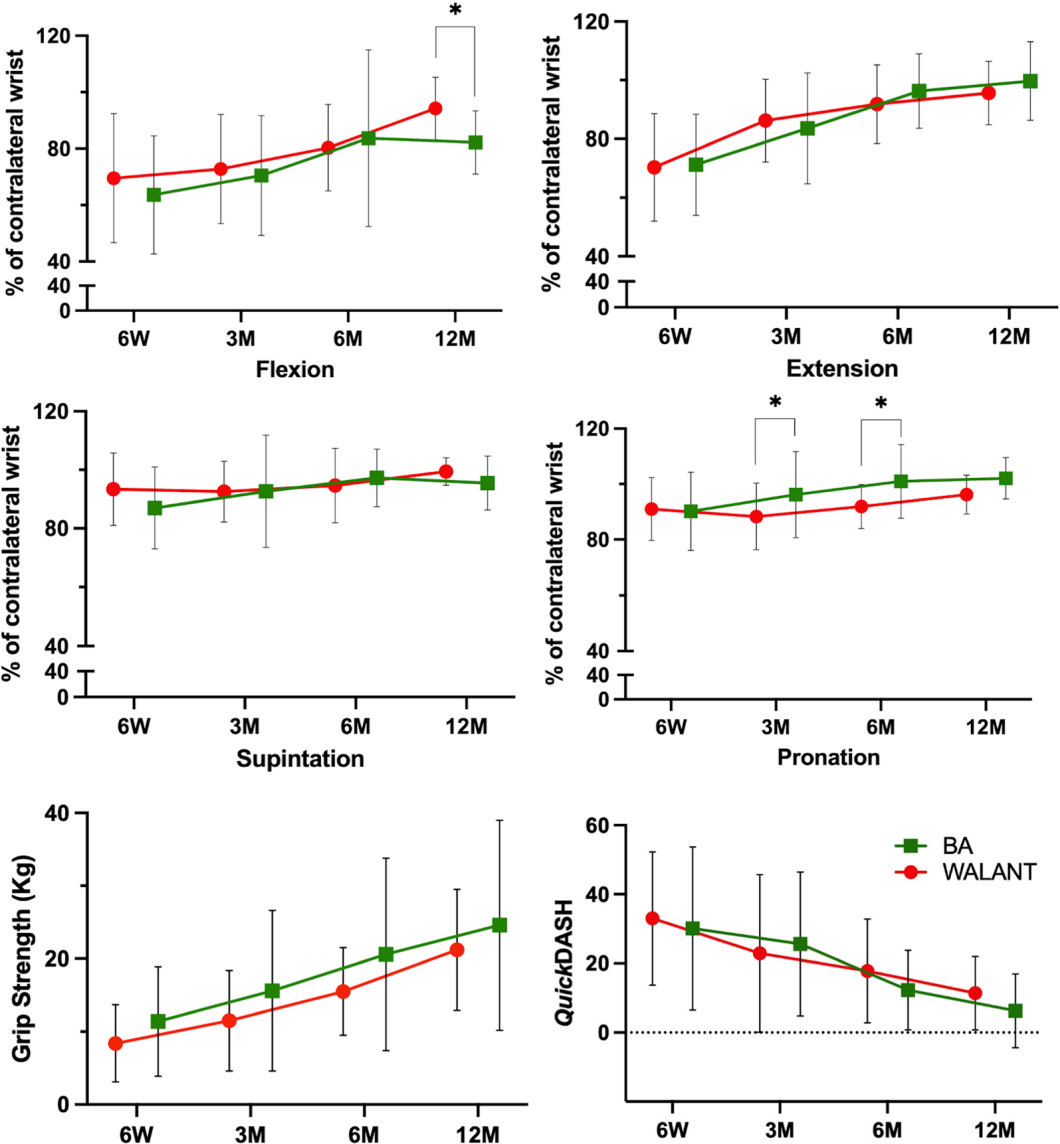
These graphs show the changes in the range of motion of the wrist and forearm and the Quick disabilities of the arm, shoulder, and hand (*Quick*DASH) scores from postoperative six weeks to 1 year in the wide-awake local anesthesia no tourniquet (WALANT) and balanced anesthesia (BA) group. The error bars represent the standard deviation, and asterisks indicate a significant difference between groups

**Fig. 3:**
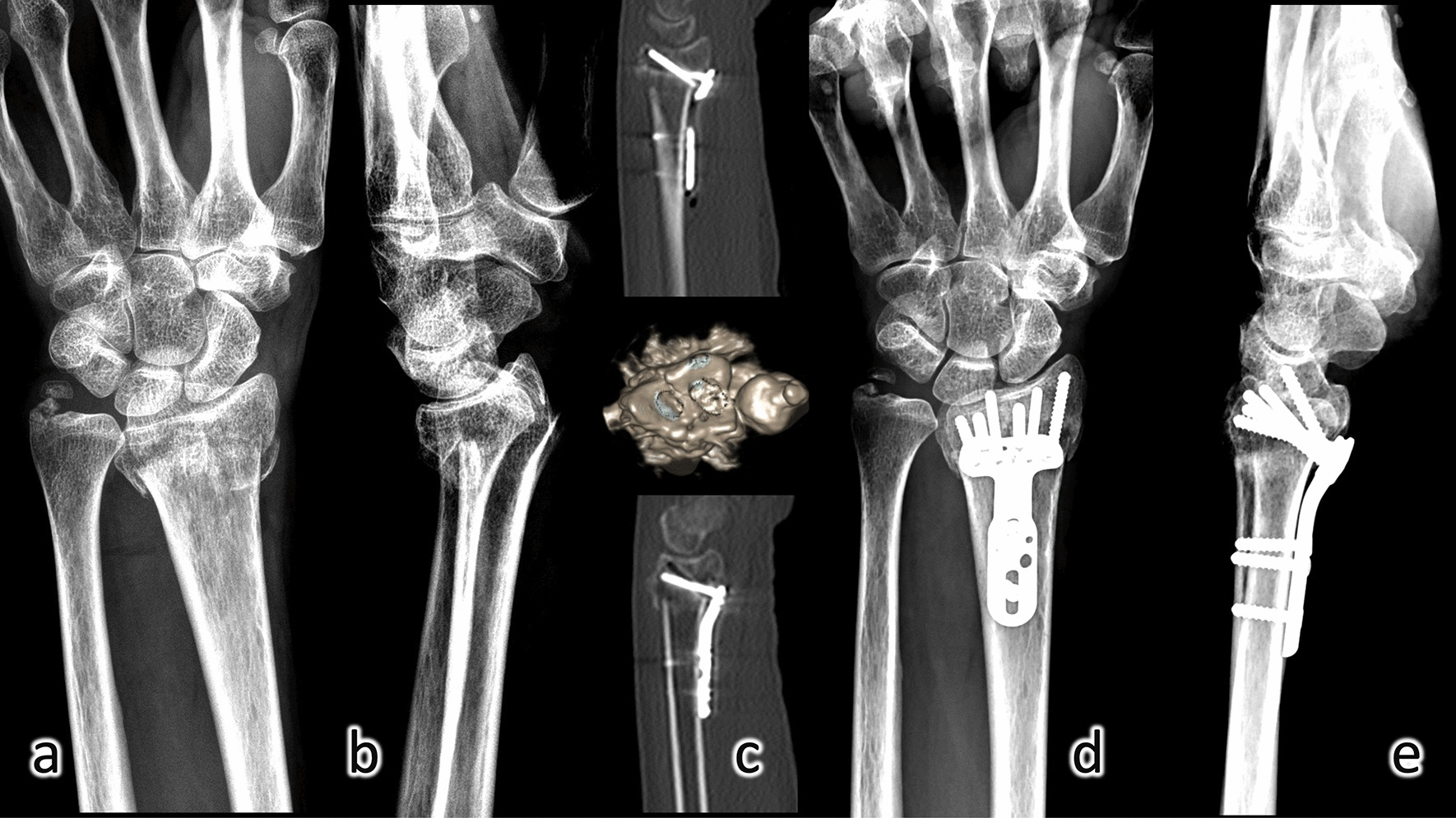
Medical images of a 61-year-old woman in radiograph of **a** posteroanterior view and **b** lateral view before operation; **c** postoperative computed tomography showed the screws captured comminuted dorsal ulnar corner fragment and anatomical reduction of the articular surface at both radiocarpal surface and sigmoid notch; radiograph of **d** posteroanterior view and **e** lateral view at 1-year follow-up postoperatively

**Fig. 4:**
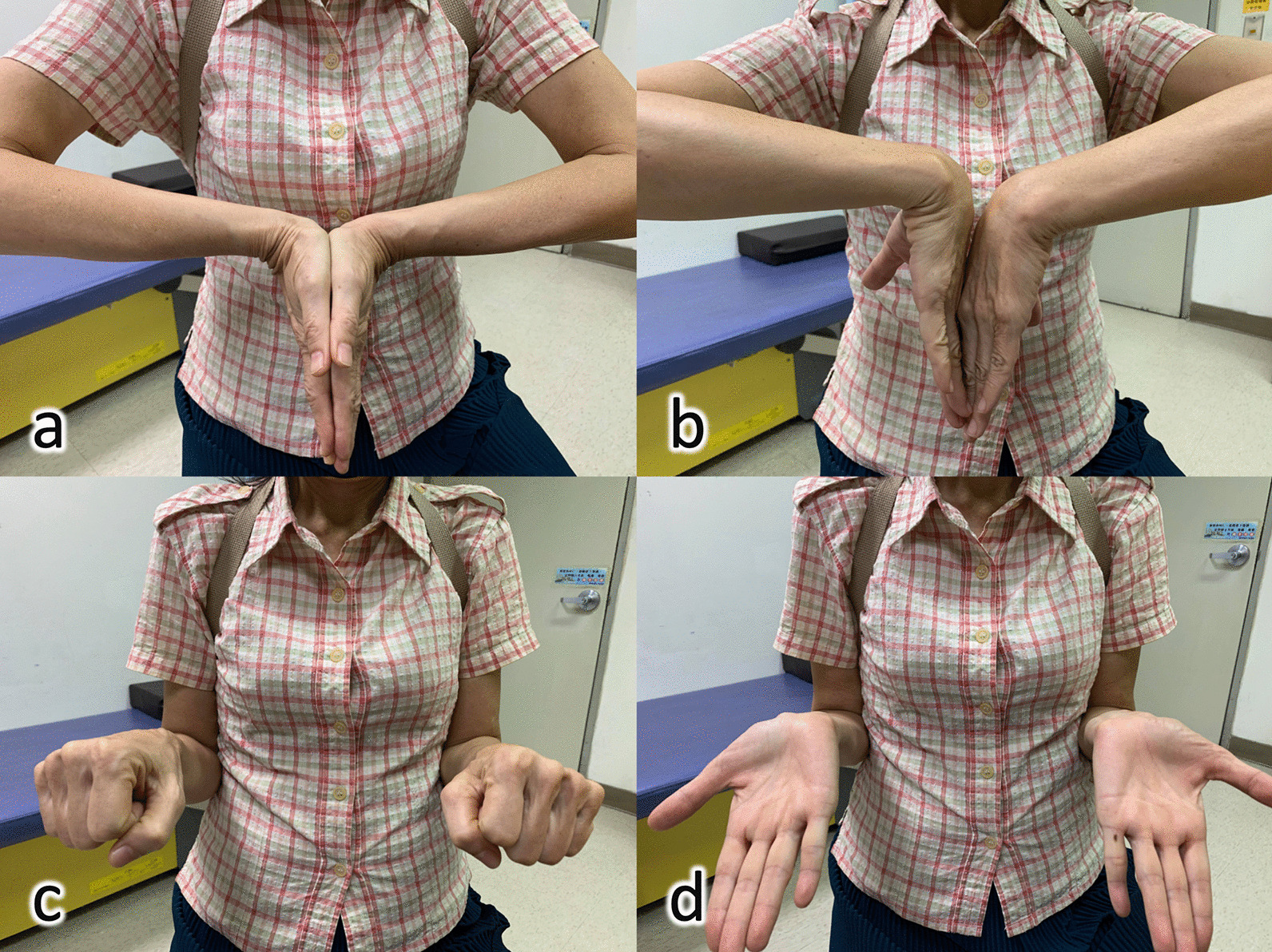
A 61-year-old woman undergoing volar plating surgery via wide-awake local anesthesia no tourniquet technique and the 1-year follow-up functional outcome of **a** wrist extension, **b** wrist flexion, **c** forearm pronation and **d** forearm supination

The postoperative 3-month pronation (88% vs. 96%; − 8.0% [ − 15.7 to − 0.2%]) and 6-month pronation (92% vs. 100%; − 9.1% [ − 17.0 to − 1.2%]) was worse in the WALANT group than the BA group. The postoperative 12-month flexion in the WALANT group was better than in the BA group (94% vs. 82%; MD, 12.0% [2.0 to 22.1%]). The rest of the outcomes, range of motion of the wrist and the forearm, grip strength, and *Quick*DASH showed no differences between groups, and the effect sizes and its 95% CIs were provided in Tables 3, 4, 5, 6. The fluctuation of the wrist and forearm range of motion, *Quick*DASH, and grip strength during the follow-ups were summarized in Fig. 2. We provided an example of a simple intra-articular fracture treated using WALANT with good reduction and near full wrist range of motion (Figs. 3 and 4).

**Table 3:** Range of motion and grip strength at 6 weeks

		WALANT			BA			MD (95% CI)	*P* value
		Injured side	Non-injured side	Percentage†	Injured side	Non-injured side	Percentage†		
Motion	Flexion	35.7 ± 9.6°	55.5 ± 14.9°	70 ± 23%	37.8 ± 17.8°	59.7 ± 14.4°	64 ± 21%	6.0% ( − 6.0 to 17.9%)	0.32
	Extension	41.3 ± 13.0°	61.3 ± 13.2°	70 ± 18%	44.3 ± 13.9°	64.0 ± 9.8°	71 ± 17%	− 0.1% (-10.6 to 8.9%)	0.86
	Supination	84.3 ± 10.9°	91.8 ± 12.1°	93 ± 12%	79.4 ± 14.8°	92.4° ± 11.7°	87 ± 14%	6.3% ( − 0.9 to 13.7%)	0.09
	Pronation	66.7 ± 10.8°	74.1 ± 8.5°	91 ± 11%	65.9 ± 13.6°	73.6 ± 12.2°	90 ± 14%	0.8% ( − 6.2 to 7.9%)	0.82
Grip strength (kg)		8.4 ± 5.3			11.4 ± 7.5			− 2.9 ( − 6.1 to 0.2)	0.65
*Quick*DASH		33.0 ± 19.3			30.1 ± 23.6			2.9 ( − 13.0 to 18.7)	0.72

^†^Injured side as percentage of noninjured side; *Quick*DASH, quick disabilities of the arm, shoulder, and hand; WALANT, wide-awake local anesthesia no tourniquet; BA, balanced anesthesia; Mean ± Standard deviation; MD, mean difference; CI, confidence interval

**Table 4:** Range of motion and grip strength at 3 months

		WALANT			BA			MD (95% CI)	*P* value
		Injured side	Non-injured side	Percentage†	Injured side	Non-injured side	Percentage†		
Motion	Flexion	41.8 ± 13.5°	58.2 ± 11.8°	72 ± 19%	41.2 ± 13.8°	59.1 ± 12.9°	70 ± 21%	2.3% ( − 9.1 to 13.8%)	0.69
	Extension	52.4 ± 10.2°	62.7 ± 11.7°	86 ± 14%	51.5 ± 13.2°	61.6 ± 9.4°	84 ± 19%	2.6% ( − 6.6 to 11.8%)	0.57
	Supination	86.5 ± 15.8°	94.5 ± 12.3°	93 ± 10%	86.0 ± 20.7°	92.6 ± 11.2°	93 ± 20%	− 0.1% ( − 8.9 to 8.7%)	0.98
	Pronation	68.1 ± 14.0°	76.8 ± 11.2°	88 ± 12%	70.3 ± 15.1°	72.9 ± 10.1°	96 ± 16%	− 8.0% ( − 15.7 to − 0.2%)	0.05
Grip strength (kg)		11.5 ± 6.9			15.6 ± 11.0			− 4.1 ( − 9.2 to 1.0)	0.11
*Quick*DASH		22.9 ± 22.8			25.6 ± 20.8			− 2.7 ( − 15.6 to 10.2)	0.67

^†^Injured side as percentage of noninjured side; *Quick*DASH, quick disabilities of the arm, shoulder, and hand; WALANT, wide-awake local anesthesia no tourniquet; BA, balanced anesthesia; Mean ± Standard deviation; MD, mean difference; CI, confidence interval

**Table 5:** Range of motion and grip strength at 6 months

		WALANT			BA			MD (95% CI)	*P* value
		Injured side	Non-injured side	Percentage†	Injured side	Non-injured side	Percentage†		
Motion	Flexion	48.8 ± 10.4°	61.6 ± 12.7°	80 ± 15%	47.4 ± 13.1°	59.4 ± 11.7°	84 ± 31%	− 3.4% ( − 21.0 to 14.1%)	0.69
	Extension	57.9 ± 8.3°	64.0 ± 10.4°	92 ± 13%	59.7 ± 8.0°	62.7 ± 9.1°	96 ± 13%	− 4.5% (-13.7 to 4.8%)	0.33
	Supination	91.3 ± 20.5°	96.4 ± 16.1°	95 ± 13%	95.8 ± 13.5°	99.1 ± 15.3°	97 ± 10%	− 2.7% (-10.7 to 5.3%)	0.50
	Pronation	76.1 ± 11.6°	82.6 ± 8.2°	92 ± 8%	74.8 ± 9.8°	74.8 ± 11.3°	100 ± 13%	− 9.1% ( − 17.0 to − 1.2%)	0.03
Grip strength (kg)		15.5 ± 6.0			20.6 ± 13.2			− 5.2 ( − 12.4 to 2.2)	0.16
*Quick*DASH		17.8 ± 15.0			12.3 ± 11.5			5.5 ( − 5.2 to 16.2)	0.30

^†^Injured side as percentage of noninjured side; *Quick*DASH, quick disabilities of the arm, shoulder, and hand; WALANT, wide-awake local anesthesia no tourniquet; BA, balanced anesthesia; Mean ± Standard deviation; MD, mean difference; CI, confidence interval

**Table 6:** Range of motion and grip strength at 12 months

		WALANT			BA			MD (95% CI)	*P* value
		Injured side	Non-injured side	Percentage†	Injured side	Non-injured side	Percentage†		
Motion	Flexion	61.3 ± 10.4°	65.6 ± 11.4°	94 ± 11%	47.8 ± 10.3°	58.3 ± 11.2°	82 ± 11%	12.0% (2.0 to 22.1%)	0.02
	Extension	66.2 ± 6.8°	69.6 ± 7.5°	96 ± 10%	63.9 ± 14.1°	63.9 ± 10.5°	100 ± 14%	− 4.0% ( − 14.8 to 6.7%)	0.44
	Supination	105.8 ± 18.9°	106.5 ± 19.3°	99 ± 5%	96.4 ± 12.8°	101.3 ± 12.7°	96 ± 9%	3.9% ( − 2.3 to 10.1%)	0.20
	Pronation	82.3 ± 12.7°	85.6 ± 12.3°	96 ± 7%	74.9 ± 10.5°	73.7 ± 11.5°	102 ± 8%	− 5.9% ( − 12.6 to 0.8%)	0.08
Grip strength (kg)		21.2 ± 8.3			24.6 ± 14.4			− 3.4 ( − 13.2 to 6.5)	0.49
*Quick*DASH		11.4 ± 10.6			6.3 ± 10.7			5.1 ( − 4.7 to 14.9)	0.29

^†^Injured side as percentage of noninjured side; *Quick*DASH, quick disabilities of the arm, shoulder, and hand; WALANT, wide-awake local anesthesia no tourniquet; BA, balanced anesthesia; Mean ± Standard deviation; MD, mean difference; CI, confidence interval

### Patient experience survey

Overall satisfaction was similar between the WALANT (8.7 ± 2.0) and BA (8.5 ± 1.7) groups (MD, 0.2 [ − 0.8 to 1.2])). In the WALANT group, patients reported low injection pain (1.7 ± 2.0), intraoperative pain (1.2 ± 1.9), and intraoperative anxiety (2.3 ± 2.8) scores. Sixty percent (17 patients) of the WALANT group had previous experience of anesthesia under sedation, but overall satisfaction was comparable between those with and without previous BA experience (8.6 ± 1.5 vs. 8.7 ± 2.4; MD, − 0.1 [ − 1.8 to 1.5]). Most patients (71.4%) in the WALANT group would choose the same technique if given the option again, and 75% would recommend it to others. A chi-square test showed no significant association between previous experience of anesthesia under sedation and patient preference for WALANT or BA in the future (*X*^2^ (d*f* = 1, *N* = 28) = 2.5, *P* = 0.11).

## Discussion

In this retrospective study, we evaluated patients who underwent distal radius plating surgery using the WALANT technique and BA. The WALANT group had fewer intra-articular DRF fractures than the BA group (AO type A/B/C: 30/3/5 vs. 26/10/19, *P* = 0.009). Both groups had comparable surgical performance, range of motion, grip strength, and *Quick*DASH scores. Overall satisfaction was also similar between groups (8.7 vs. 8.5; MD, 0.2 [ − 0.8 to 1.2]). Patients in the WALANT group reported tolerable pain (1.2 ± 1.9/10) and anxiety (2.3 ± 2.8/10), with 75% willing to choose WALANT again.

The overall surgical time was shorter in the WALANT group. However, this conclusion may be biased because the fracture characteristics were different between the WALANT and BA groups. According to our experience, the WALANT technique is preferred for patients with relatively less complex fracture patterns. Patients with a more complex fracture pattern inherently have a longer duration of surgery, making BA more appropriate.

The mean surgical time for the WALANT group was 71.5 min versus 91.3 min for BA (MD, − 19.8 [ − 33.8 to − 5.9]; *P* < 0.01). The surgical time was calculated from the beginning of the incision to the end of wound closure. It is important to note that the time required to establish WALANT, which takes 20–25 min for the onset of the hemostatic effect of epinephrine [10, 20], was not included in the reported surgical time in the study. To make the whole procedure efficient, surgeons usually did the surgical prep while waiting for the hemostatic effect of epinephrine to work. Overall, the actual surgical time might be slightly longer than recorded in the WALANT group.

As shown in Table 1, the characteristics of DRFs in the WALANT group were different—AO/OTA class 2R3A accounted for 78.9% of patients in the WALANT group and only 47.2% of patients in the BA group. When patients with more complex fracture patterns, with a suspected longer operation time, surgeons would try to provide BA as an option for patients. Regarding blood loss, the WALANT group was higher than the BA group. The application of a tourniquet and the sedative effect of the anesthetics, which led to the low mean arterial pressure in the BA group, may have contributed to the result.

Dukan et al. asserted that one of the advantages of WALANT is that it allows the patient to stay awake during the operation, which enables the surgeon to assess the DRF in real-time and ensure the stability of fixation with no mechanical obstruction of the tendon by the implant [21]. However, despite this intuitive advantage, no difference in functional outcomes were observed in this study, which found that the overall reduction quality was similar between the two groups. It has been speculated that using WALANT may paradoxically affect patients undergoing hand surgery, as they are awake and able to participate in the surgical process, which may lead to increased anxiety. It has also been suggested that surgeons tend to be gentler during fracture reduction with WALANT than when a patient is sedative. However, this claim is purely speculative and requires further investigation.

The WALANT technique provides local anesthetic effects only to the operative area. The preoperative plan for surgery must be meticulous since there is less room for intraoperative changes. For example, one of the cases in the WALANT group experienced an intraoperative iatrogenic fracture. As a result, the incision had to be extended proximally for a longer plate. Since the maximum dose of lidocaine had been reached, an anesthesiologist was consulted to complete the surgery under sedation and multimodal pain control. WALANT is more suitable in patients with simpler fracture patterns, and good preoperative planning is critical in achieving favorable results. We recommend not treating complex extra-articular or intra-articular fractures using the WALANT technique. In areas needing more specialized anesthesiologists and financial resources for GA, the WALANT method allows the surgeon to perform the surgery independently at a lower cost [22].

While the SANE scale has been utilized in previous hand surgery literature, it has been noted that it is a general assessment that primarily focuses on the process of care rather than the result of care. However, the results and process of care are equally important, as the feedback given by patients when they return to their daily lives contains more objectively valuable factors which genuinely reflect the impact of the surgery on them, whether positive or negative. Many studies focused on satisfaction with the WALANT technique; however, most are soft tissue and minor procedure [23–25], and only one clinical trial evaluated patient satisfaction and showed significantly higher satisfaction with WALANT than with GA [5]. However, we found no significant difference in the overall surgical experience between patients in the WALANT and BA groups (8.7 vs. 8.5; MD, 0.2 [ − 0.8 to 1.2]). Similarly, no difference was found between patients with and without previous experience with BA in the WALANT group (8.6 vs. 8.7; MD, − 0.1 [ − 1.8 to 1.5]). While under WALANT, patients would experience fewer episodes of postoperative nausea and vomiting, and more extended hospital stays; they have to endure the procedure while awake. Especially among patients who are easily made anxious, it can be a traumatic experience regardless of preoperative education.

The patient experience survey was performed during the postoperative visits to the clinic. Most patients rated their experience as “highly satisfactory.” The most cited reason patients were satisfied with the WALANT technique was that it allowed them to be discharged immediately after the surgery without being observed at the PACU. Second, patients with previous experience in BA reported a shorter recovery time from anesthesia and fewer side effects, such as nausea and vomiting. Finally, some elderly patients stated that WALANT was a suitable alternative to BA because they were concerned with the risks associated with sedation.

On the other hand, for those who would not choose WALANT again, their most concerning aspect was the feeling of anxiety during the surgery. Reducing fractured bone and the sound of bone screwdrivers made the patients nervous. Establishing a trusting physician–patient relationship, playing music, or having a distracting conversation can keep the patients at ease, optimizing the overall experience in surgery via the WALANT technique [26].

This study has some limitations. Firstly, shared decision-making determined patient selection, which may have introduced selection bias and impacted the observed differences between groups. Those who were more anxious preoperatively might choose BA rather than the WALANT technique [27]. Secondly, our study did not differentiate between cases that underwent a volar approach and those that underwent a dorsal approach for fracture exposure and fixation. In addition, we avoid patients requiring plating with multiple incisions in the WALANT group. Given the inherent differences in surgical approach and potential variations in postoperative outcomes between volar and dorsal procedures, this could have biased our results. Additionally, the study's retrospective nature lacked a priori power analysis, leading to statistical power and Type II error concerns. We intentionally did not calculate post-hoc power as determined by the *P*-value [28], and all non-significant findings are underpowered in the null hypothesis significance testing framework. However, using confidence intervals can assist in determining whether a meaningful difference exists [29]. We added confidence intervals for each reported difference, including patient satisfaction, with a mean difference of 0.2 with a 95% CI of -0.8 to 1.2. While values at the tails of the interval were not clinically meaningful, the threat of a Type II error was diminished.

## Conclusion

WALANT has received much praise, but it has some limitations. Regarding the statistical results, patients’ overall satisfaction, reduction quality, and functional outcomes were comparable between the WALANT and BA groups. With meticulous preoperative planning, the WALANT technique could be an alternative to BA for DRF plating surgery in selected patients.

### Supplementary Information

**Additional file 1:** Patient Experience Survey.

## Data Availability

The datasets used and/or analyzed during the current study are available from the corresponding author on reasonable request.

## Abbreviations

BA: Balanced anesthesia
DRF: Distal radius fractures
GA: General anesthesia
WALANT: Wide-awake local anesthesia with no tourniquet
*Quick*DASH: Quick disabilities of the arm, shoulder, and hand
CIs: Confidence intervals
PACU: Post-anesthesia care unit
AO/OTA: AO Foundation/Orthopaedic Trauma Association
SANE: Single Assessment Numeric Evaluation
MD: Mean difference
