# Supplementary material for: Comparison of distal radius fracture plating surgery under wide-awake local anesthesia no tourniquet technique and balanced anesthesia: a retrospective cohort study
Source: J Orthop Surg Res. 2023 Oct 3;18:746. doi: 10.1186/s13018-023-04243-0 (PMC10546761; doi:10.1186/s13018-023-04243-0)
Supplement: Supplementary file 1 — Additional file 1: Patient Experience Survey. [file 13018_2023_4243_MOESM1_ESM.pdf]

# Patient Experience Survey on the Use of WALANT in Distal Radius Fractures

| | | | |
|---|---|---|---|
| Name： | Chart number： | | Note |
| 1. Have you ever had surgery with anesthesia under sedation? | □ Yes | □ No | |
| 2. Have you ever had surgery via the WALANT technique before this surgery? | □ Yes | □ No | |
| 3. How much pain did you experience when the anesthesia was injected? (On a scale of 0 to 10) | | | |
| 4. How much pain did you experience during the surgery? (On a scale of 0 to 10) | | | |
| 5. How anxious did you feel during the surgery? (On a scale of 0 to 10) | | | |
| 6. Did you experience discomfort after the surgery? | □ Yes | □ No | |
| 7. If given a choice again, would you choose anesthesia under sedation or WALANT for the surgery? | □ WALANT | □Anesthesia under sedation | |
| 8. What is the reason for your choice in the previous question? | □ Not requiring hospitalization<br>□ Not requiring the use of a tourniquet<br>□ Not having to bear the risk of sedation | | Other: |
| 9. Would you recommend this type of anesthesia to your family and friends? | □ Yes | □ No | |
| 10. Overall satisfaction with WALANT surgery? (On a scale of 0 to 10) | | | |
